# Preliminary characterization of rectification for transradial prosthetic sockets

**DOI:** 10.1038/s41598-024-56333-6

**Published:** 2024-03-08

**Authors:** Calvin C. Ngan, Vishal Pendse, Harry Sivasambu, Elaine Ouellette, Neil Ready, Jan Andrysek

**Affiliations:** 1 University of Toronto, Toronto, Canada; 2 Holland Bloorview Kids Rehabilitation Hospital, Toronto, Canada

**Keywords:** Biomedical engineering, Translational research

## Abstract

Achieving proper socket fit is crucial for the effective use of a prosthesis. However, digital socket design lacks standardization and presents a steep learning curve for prosthetists. While research has focused on digital socket design for the lower-limb population, there is a research gap in upper-limb socket design. This study aimed to characterize the design (rectification) process for the transradial socket, specifically the three-quarter Northwestern-style design, towards the development of a more systematic, data-driven socket design approach. Fourteen (n = 14) pairs of unrectified and rectified plaster models were compared. Six common rectification zones were identified through shape analysis, with zones of plaster addition being the most prominent in terms of volume and surface area. A novel 3D vector mapping technique was employed, which revealed that most of the shape changes occurred in the anterior–posterior and proximal–distal directions. Overall, the interquartile range of each rectification zone demonstrated reasonable consistency in terms of volume, surface deviation, and 3D vector representation. The initial findings from this study support the potential for quantitively modelling the transradial socket design process. This opens the door for developing tools for categorizing and predicting socket designs across diverse populations through the application of techniques such as machine learning.

## Introduction

The prosthetic socket serves as an interface between the human body and the prosthesis. Proper socket fit is crucial towards enabling effective prosthetic function and positive rehabilitation outcomes^1^. A well-fitted socket must facilitate effective force transferring, prevent tissue damage, minimize user discomfort, and allowing use and agency over the prosthesis^2^. In practice, the determination of socket fit relies on user-reported feedback about comfort and function, as well as prosthetists’ visual examination of the residual limb after diagnostic socket fitting^3,4^. The examination includes observing the blanching of the skin, tissue bulging, and residual limb’s range of motion. This highlights the field of prosthetics is largely practiced in a non-quantitative manner.

Custom sockets are fabricated by first capturing the shape of the residual limb, either using plaster of Paris bandages or optical scanning. The resulting model (i.e., either a physical positive plaster model or a digital one) then needs to be rectified, which is the process of adding or removing material in specific anatomical regions. Rectification is a crucial step in establishing an optimal residual limb–socket interface, ensuring the design meets the key criteria of a well-fitted socket listed above. Following diagnostic socket fitting and clinical assessment, the socket shape is finalized, and the socket is then fabricated based on this model^4,5^. While the rectification process is essential for achieving a comfortable and well-fitting prosthesis, it is largely considered an art form that has not been well documented in literature. Objectively characterizing this rectification is an important step towards improving and standardizing the socket design process since existing practice is subjective and outcomes (in terms of socket fit) can vary depending on factors such as the experience level of the practicing prosthetist^6^.

Quantification of the geometry to inform the device shape design has been pursued for lower-limb applications^7–11^ and orthotics^12^; however, no such work exists for upper-limb prosthetics. While lower-limb and upper-limb sockets share similar design principles, their specific requirements are distinct. Upper-limb sockets, including at the transradial level, must continuously overcome the effect of gravity, making suspension control a crucial element in their design^13^. Thus, the socket shape is critical to concurrently achieve comfortable fit and good suspension characteristics, as well as maximizes the contact surface area and ensures good skin-to-electrode contact for myoelectric prostheses^14,15^. One of the most widely used transradial socket designs is the Northwestern-style socket, owing to its intimate fit and self-suspending characteristics^16^. Northwestern-style sockets achieve suspension by relying on supracondylar suspension techniques, whereby the socket extends proximally to the humeral condyles and compresses the tissue in the medial–lateral plane superior to the epicondyles^17^. However, due to the limitations in the design, such as limited range of motion at the elbow and excessive heat and perspiration inside the socket, the olecranon region of the socket is eliminated, termed the three-quarter design^18^.

Despite increased interest within the clinical community, socket design lacks standardization and presents a significant learning curve for the prosthetist^19^. In fact, socket rectification is known to be one of the most arduous and time-consuming tasks to master in prosthetic design^8^. This is particularly true for the transradial population; given its relatively small population size, it is widely acknowledged that prosthetists often lack the opportunity and familiarity to serve the highly complex fitting needs of this group^20^. However, it may be possible to address this major limitation by exploring and establishing models that characterize the unrectified-to-rectified socket shape changes. Therefore, the objective of this study was to characterize the rectification process of the three-quarter Northwestern-style transradial socket by analyzing the shape variations between pairs of unrectified and rectified positive plaster models. This work can lead to a more comprehensive understanding of the rectification process, consequently laying the groundwork for a more systematic, data-driven approach towards digital socket design.

## Results

A total of 14 participants were recruited between December 2020 to June 2023 (Table 1), resulting in the collection of 14 pairs of unrectified and rectified models for analysis. The volume difference between each pair of unrectified and rectified models was examined and is shown in Table 2.

**Table 1:** Participant characteristics.

	Sex	Cause of limb absence	Side	Age (years)	Qualitative description		
					Length	Shape of residual limb	Tissue consistency
P1	F	Congenital	L	11	Short	Conical	Moderate
P2	M	Congenital	L	5	Short	Conical	Moderate
P3	M	Acquired	L	74	Long	Conical	Moderate
P4	M	Congenital	L	14	Short	Round	Moderate
P5	F	Congenital	R	14	Short	Conical	Firm
P6	M	Congenital	R	53	Short	Conical	Firm
P7	M	Acquired	R	13	Short	Round	Soft
P8	F	Congenital	L	6	Short	Round	Moderate
P9	M	Congenital	L	43	Long	With bulbous end	Firm
P10	F	Congenital	L	22	Short	Slender	Moderate
P11	F	Congenital	L	8	Short	Round	Moderate
P12	M	Congenital	L	7	Short	Conical	Moderate
P13	F	Acquired	L	16	Short	Round	Soft
P14	F	Congenital	L	7	Long	Slender	Moderate

**Table 2:** Global volume difference between each pair of unrectified and rectified models.

	Unrectified volume (mL)	Rectified volume (mL)	Difference % relative to the unrectified
P1	276	292	+ 6%
P2	228	264	+ 16%
P3	686	750	+ 9%
P4	444	478	+ 8%
P5	433	453	+ 5%
P6	559	590	+ 6%
P7	645	646	0%
P8	221	231	+ 5%
P9	934	1045	+ 12%
P10	228	234	+ 3%
P11	238	261	+ 10%
P12	269	295	+ 10%
P13	600	606	+ 1%
P14	419	480	+ 15%
Mean (1 SD)	441 (219)	473 (238)	+ 7% (5%)

One standard deviation is denoted as 1 SD (mL).

### Examining the changes in cross-sectional area along the length of models

**Figure 1:**
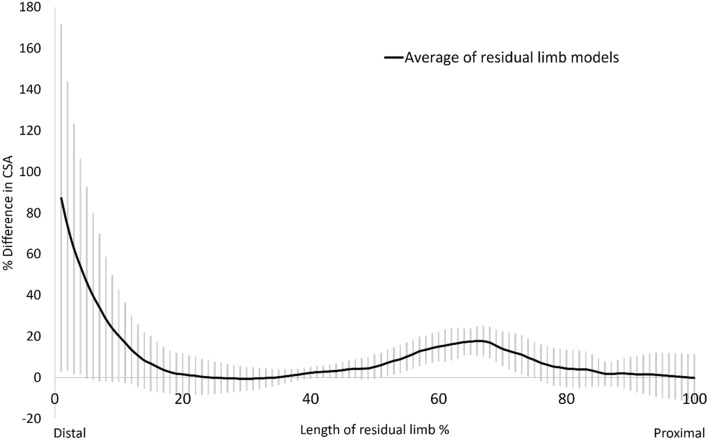
Mean differences in CSA between unrectified and rectified models along the length of the reference axis. Shaded bands represent one standard deviation. Positive values denote the mean increase in CSA in the rectified model relative to the unrectified model, while negative values indicate the mean reduction in CSA.

Figure 1 illustrates the differences in cross-sectional area (CSA) between unrectified and rectified models along the length of the residual limb. A trend was observed across all pairs, where positive values were found at both the distal end and in the middle to proximal portion of the model, indicating the addition of plaster; thus, volume increases in the socket were concentrated in these regions during rectification.

### Identifying and characterizing localized rectification zones

**Figure 2:**
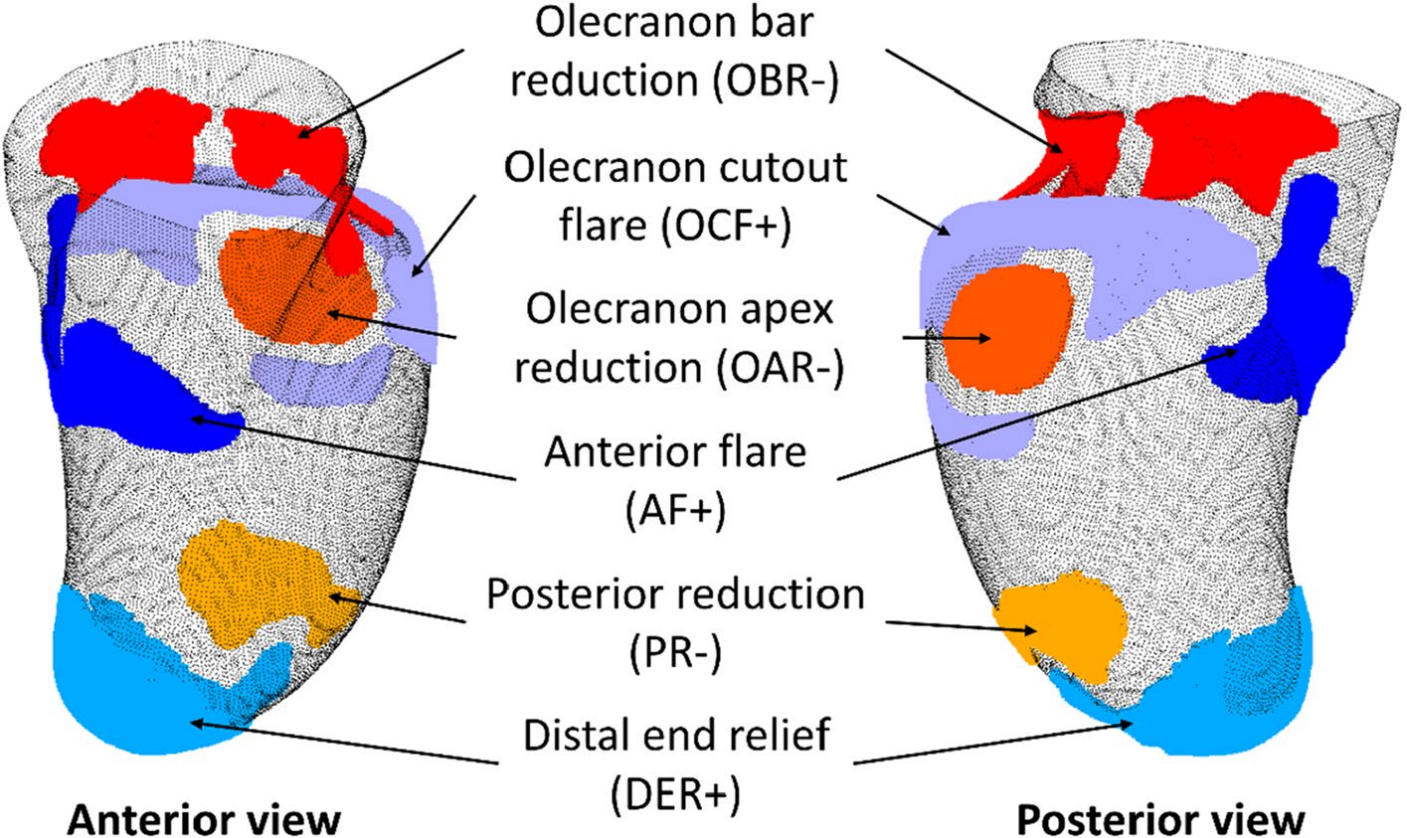
Common rectification zones found in the transradial socket design. The “+” and “−” signs at the end of each rectification zone denote an addition or reduction in volume, that is, the addition or removal of plaster, respectively.

Six common rectification zones were found in the design of the three-quarter Northwestern-style transradial socket (Fig. 2). Based on the relevant literature and clinical input of two experienced prosthetists (E.O. and N.R.), these zones were labelled as (1) olecranon bar reduction (OBR), (2) posterior reduction (PR), (3) anterior flare (AF), (4) olecranon cutout flare (OCF), (5) olecranon apex reduction (OAR), and (6) distal end relief (DER).

- OBR: Reductions at the region superior to the olecranon process. A significant amount of plaster is removed to enable the socket to apply compression at the triceps tendon, providing suspension and stabilizing the socket on the residual limb^17^.
- PR: Reductions in the posterior–distal part of the rectified model. While the majority of tissue compression is achieved during casting, prosthetists may perform minor shaping adjustments in the PR region during rectification to further compress the underlying tissue to facilitate an intimate fit between the socket and residual limb. It should be noted that PR was only observed in short residual limbs but was absent in long residual limbs. This may be attributed to the bony nature of the long residual limbs included in this study, which had limited redundant tissue distal to the epicondyles. Additionally, long residual limbs naturally possess larger surface contact areas to facilitate effective socket suspension; thus, no further volume reduction was needed in the PR during rectification.
- AF: Addition in the anterior part of the rectified model to create the trimline of the socket. The trimline is specifically flared outward to prevent tissue irritation and impingement^17^. The placement of the lowest point of the trimline depends on the length of the residual limb. In the case of longer residual limbs, the lowest point is extended towards the distal end of the socket to increase elbow flexion. In contrast, for short residual limbs, the lowest point of the trimline is positioned at the cubital fossa in order to maximize surface contact area, which improves socket suspension.
- OAR and OCF: Reduction and addition in the posterior–proximal part of the rectified model, respectively, to create an obturator opening in the posterior–proximal quadrant of the socket, known as the three-quarter socket design^18^. During rectification, material is removed from the olecranon apex while plaster is added in the OCF region to create a flared trimline around the olecranon. This design is known to improve ventilation inside the socket, which reduces perspiration, leading to improved comfort for the client and socket suspension. In addition, this feature can also facilitate a greater range of motion for the elbow compared to an enclosed socket design.
- DER: Addition at the distal end of the rectified model. Plaster is added to increase volume and the length of the socket. Its purpose is to alleviate pressure and prevent the socket from impinging on the residual limb, particularly during activities that involve pushing. Furthermore, for the paediatric population, the added volume also serves to accommodate growth.

**Figure 3:**
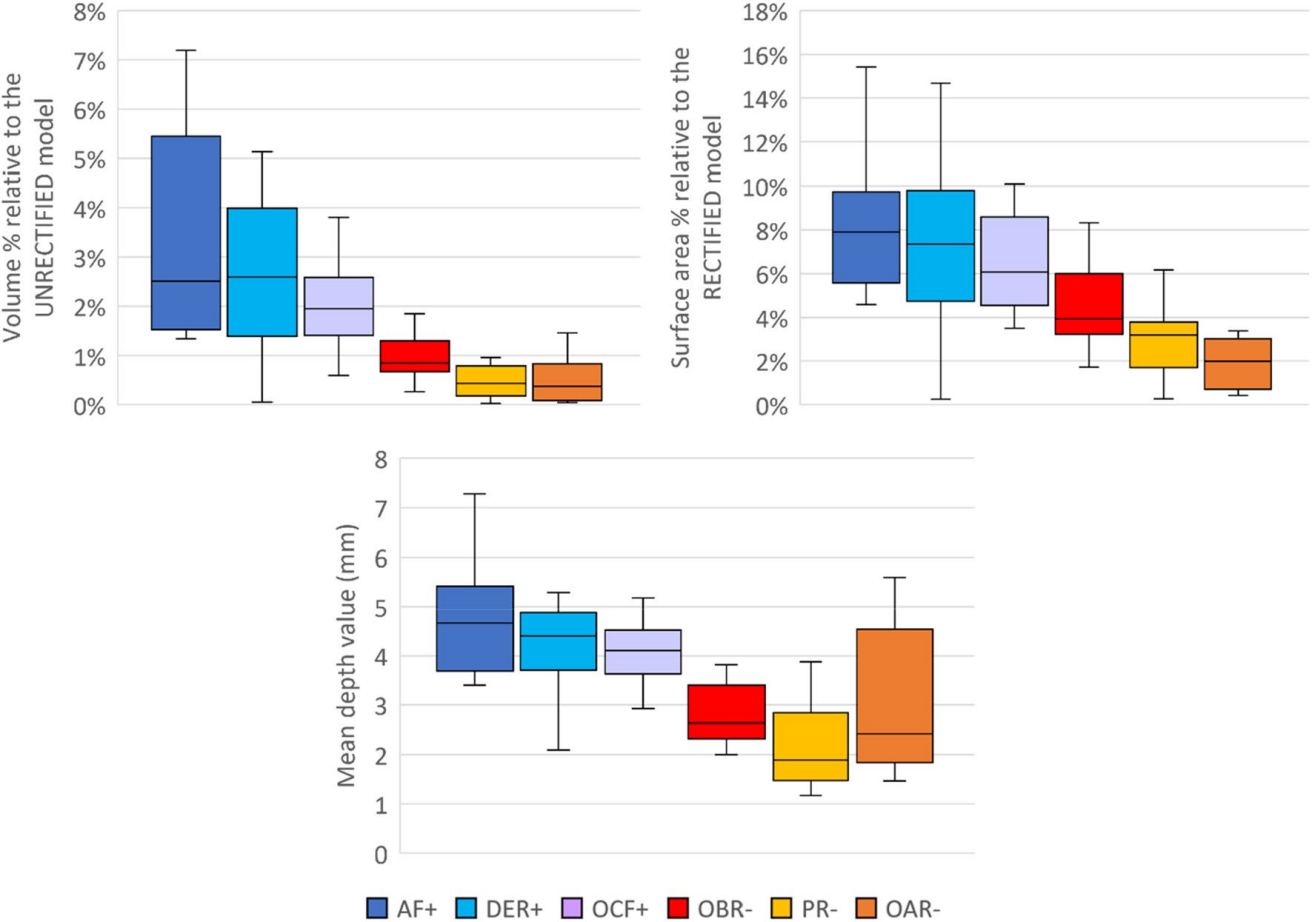
Box plots showing the volume %, surface area %, and mean depth value of each rectification zone. The “+” and “−” signs at the end of rectification zone indicate plaster addition or removal, respectively.

Surface area, volume, and mean depth value were calculated for each rectification zone (Fig. 3). Surface area % is presented relative to the rectified model, as the rectification zones were identified from the rectified models. Conversely, volume % is presented relative to the unrectified model, indicating an addition or removal of plaster to the model during the rectification process. Interestingly, all three graphs demonstrated similar trends among all zones, where rectification zones related to plaster addition (AF, DER, and OCF) were the most prominent, while rectification zones related to plaster reduction (OAR and PR) had the smallest magnitude.

**Figure 4:**
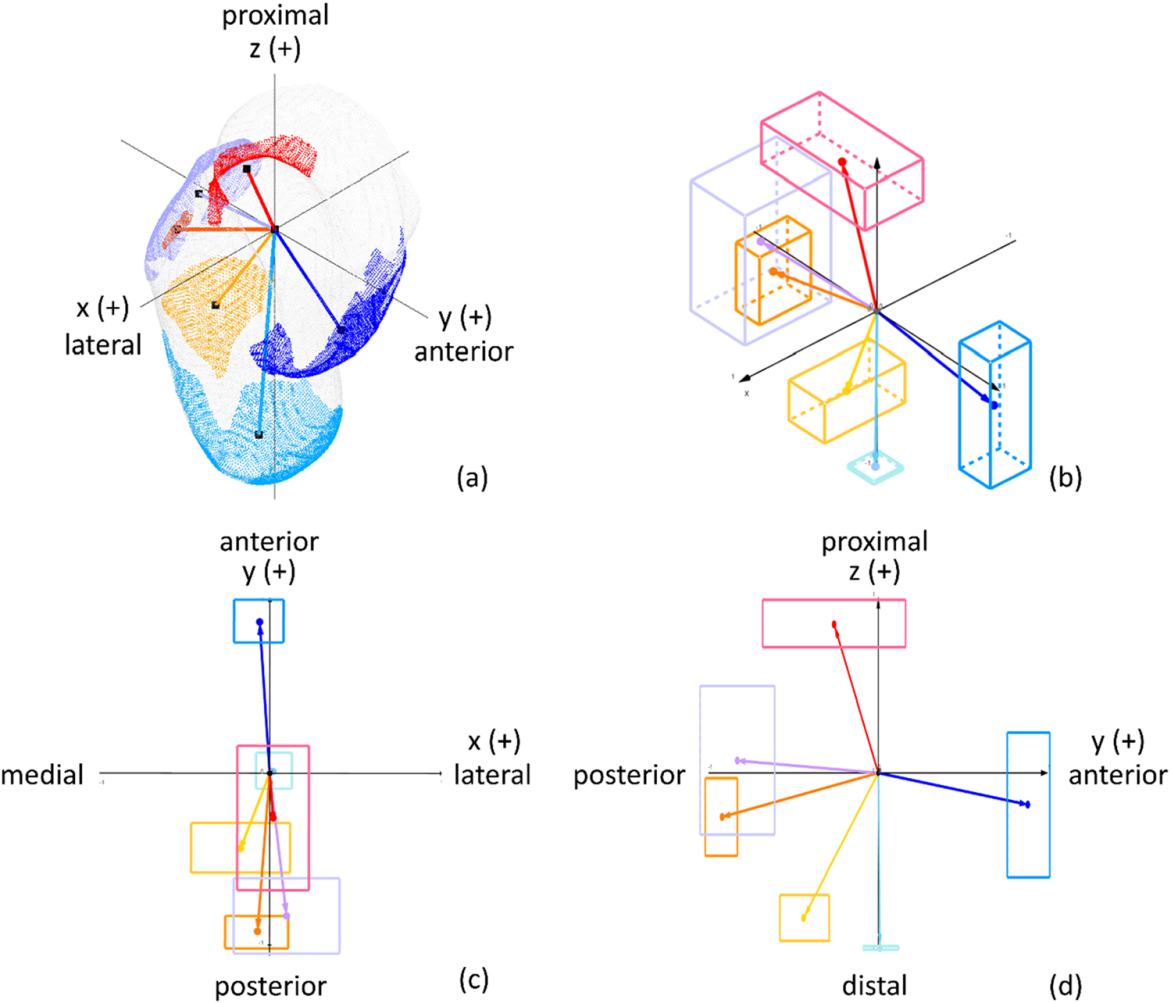
Normalized average vectors of each rectification zones: (**a**) example of vectors extending from the origin to the centroid of each rectification zone in a rectified model; (**b**) average vectors of each rectification zone presented in a unit circle, where the boxes represent one standard deviation in each axis; (**c**) unit vectors viewed in the x–y plane; and (**d**) unit vectors viewed in the y–z plane.

**Figure 5:**
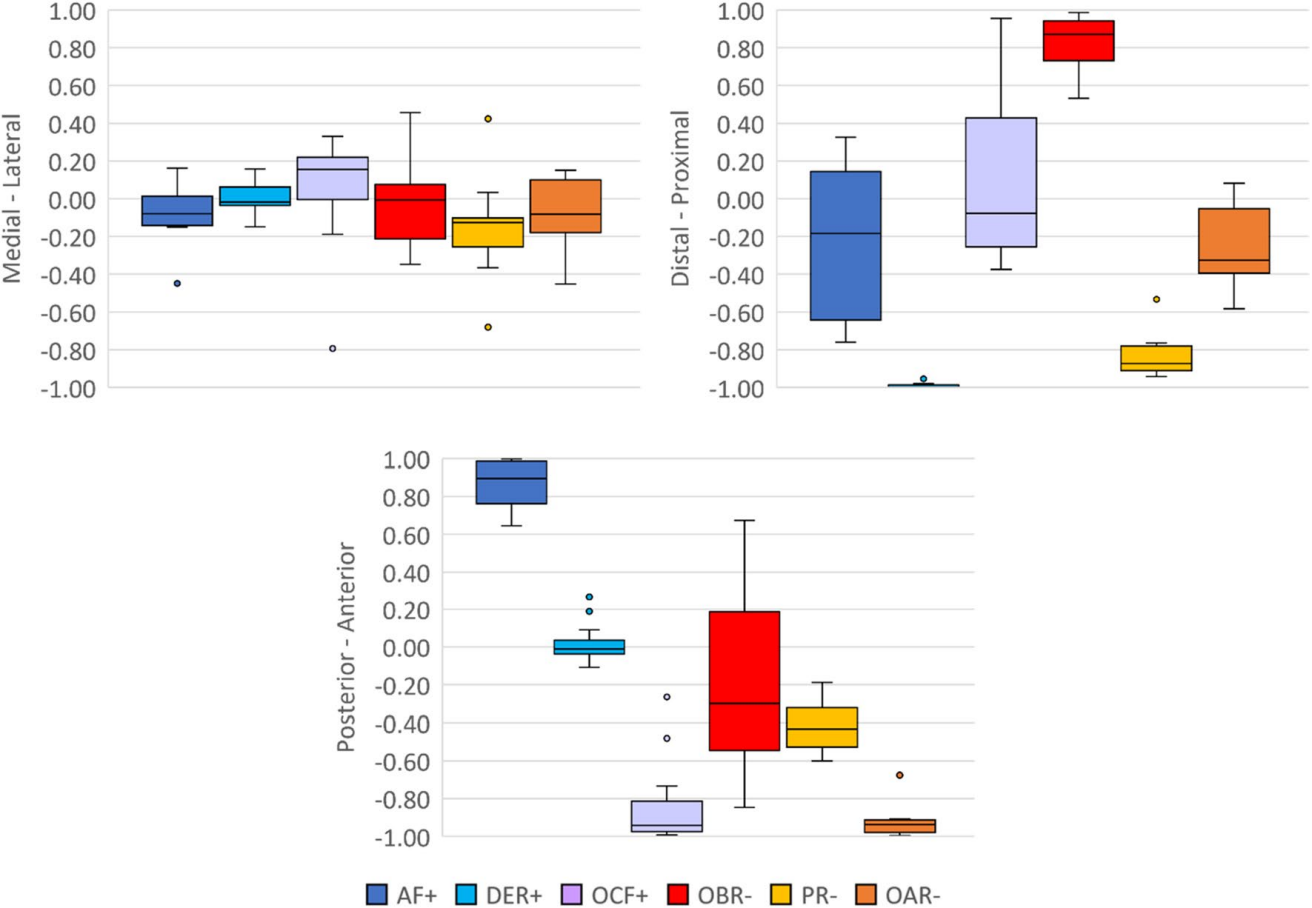
Box plots showing the variability of the unit vector of each zone in the medial–lateral (x), anterior–posterior (y), and proximal–distal (z) directions.

To quantitatively map the locations of rectification zones within the rectified model, the average location of each zone within the anatomical landmark-based coordinate convention was calculated and expressed in 3D unit vectors (Table 3). Figure 4 illustrates the location of each rectification zone; part c and d present the vectors in the frontal and sagittal plane, respectively, serving as supplementary presentation to enhance comprehension of the 3D figures. It can be observed that most of the shape changes occurred in the anterior–posterior and proximal–distal directions. However, it should be noted that the directions of these vectors could be affected by certain factors, including the length of the residual limb. As shown in Fig. 5, the x-component of each zone predominantly fell within the range of -0.26 to 0.19, indicating less variance in the medial–lateral direction.

**Table 3:** Average unit vector for each rectification zone.

	x	y	z
AF	− 0.10 (0.17)	0.87 (0.12)	− 0.20 (0.41)
DER	0.00 (0.09)	0.02 (0.10)	− 0.99 (0.01)
OAR	− 0.07 (0.18)	− 0.92 (0.09)	− 0.25 (0.22)
OBR	− 0.02 (0.22)	− 0.24 (0.44)	0.84 (0.13)
OCF	0.06 (0.29)	− 0.85 (0.22)	0.06 (0.41)
PR	− 0.16 (0.30)	− 0.42 (0.13)	− 0.83 (0.13)

One standard deviation in parentheses.

## Discussion

Healthcare is undergoing a transformative shift towards digitally driven, data-centric solutions across various domains^21^. In the field of prosthetics, research has focused on quantifying the conventional manual rectification process for lower-limb sockets, either by examining the consistency of rectification or quantifying the shape difference between unrectified and rectified models^9–11^. These works have demonstrated the potential for implementing data-driven methods in digital socket design^7,22,23^. Building upon these research efforts, this paper is the first to characterize the rectification process of transradial socket design. It is recognized that sockets are highly personalized devices tailored to meet the needs of an individual, and thus, no two sockets are identical. While the small sample size precludes the formulation of definitive conclusions, this preliminary study has revealed general trends and design elements in transradial sockets, and quantified their characteristics in terms of volume, surface deviations, and 3D vector representations.

Globally, we found a net addition of plaster during the rectification process, as the volumes of the rectified models exceeded those of the unrectified ones. This trend was consistent with the cross-sectional area (CSA) analysis, where positive CSA differences were observed along the length of the models. In terms of localized rectifications, these volume additions were primarily attributed to rectification zones such as anterior flare (AF) and distal end relief (DER). The greatest volume addition was observed in AF, as prosthetists aimed to create a more pronounced flare along the anterior trimline of the socket to prevent tissue impingement during elbow flexion and to facilitate the donning and doffing of the socket. Further, plaster was consistently added at the distal end of the socket for all types of residual limbs, which is reflected in the large DER values. There are a few reasons for this. Generally speaking, residual limbs with bulbous ends are accommodated with greater volume additions at the DER to prevent the soft tissue from bearing pressure. Similarly, when bony prominences are present, greater volume additions are also necessary to eliminate pressure points. Moreover, for short and rounded residual limbs, prosthetists often modify the sockets into a conical shape by applying compression to the soft tissue. This helps to create load-bearing surfaces and improve socket suspension. As a result, a slight elongation of the socket, that is, volume addition at the DER, is often incorporated to accommodate tissue displacement.

Supracondylar reduction plays an important role in the self-suspension and stability of the socket on the residual limb. Hence, a more aggressive approach (more material removal) for olecranon bar reduction (OBR) region might have been expected. One possible explanation is the influence of the conventional shape-capture process. During shape capture (i.e., applying the plaster bandage), prosthetists purposely apply pressure and compress key regions of the residual limb, primarily in the supracondylar and posterior–distal regions (the middle section of the residual limb)^24^. This manipulation effectively serves as a preliminary step in the socket-design process aimed at altering the limb shape towards the desired rectified form, and is not reflected as a volumetric change during rectification^5^. In contrast, AF, DER, and olecranon cutout flare (OCF) were not initially formed during shape capture and only integrated during rectification. It can, therefore, be observed that their variabilities were higher when compared to OBR. This variability can likely be attributed to the unique characteristics of individual residual limbs, such as their length, tissue consistency, and shape. It is important to note that the initial compression on the residual limb was not assessed in our study, as only comparisons of the unrectified and rectified models were undertaken. To fully understand and characterize the design of the three-quarter Northwestern-style transradial socket, future work should focus on quantifying the conventional shape-capture process by comparing the shape of the residual limb to the corresponding unrectified model. Additionally, it should be noted that the final socket shape may differ slightly from the rectified model, as it is not uncommon for prosthetists to make minor adjustments to the socket shape during the diagnostic socket fitting stage. Where more substantial shape adjustments are needed, the prosthetists will recast and repeat the rectification process; this was not necessary for any of the participants in this study, ensuring that the rectified model and final socket shapes were very similar. However, future work should consider the shape changes that can happen at the final stages, and develop and apply quantitative methods to assess them.

The analysis of the average location of the rectification zones revealed notably smaller degrees of shape changes in the medial–lateral direction compared to the anterior–posterior direction. This may appear contradictory to the design principles of Northwestern-style transradial sockets, which are known to rely on compression in the medial–lateral plane proximal to the epicondyles for socket suspension and stability^16,20^. However, as aforementioned, the initial shape of the socket, particularly the medial–lateral compression on the supracondylar region, is largely determined during the shape-capture process. Additionally, the fact that most rectifications are concentrated in the anterior–posterior direction could be attributed to accommodating for the motion of the elbow, which primarily involves flexion and extension in the sagittal plane. Therefore, it is crucial to establish a good interface between the socket and residual limb in the anterior–posterior direction to facilitate efficient load transfer and socket stability^16^. Furthermore, features such as AF and OCF are incorporated to prevent restricting the range of motion of the elbow and tissue impingement.

Examining the shape variations of the unrectified and rectified positive plaster models revealed common and consistent rectification features and patterns. These features were quantified by their volumes, surface areas, and surface deviations, offering an objective and quantitative understanding of the design principles of the three-quarter Northwestern-style transradial socket. The preliminary findings in this study suggest that the quantitative modelling of the transradial socket design process might be feasible. A study by Sanders et al.^25^ examined factors distinguishing a good socket from an oversized socket in individuals with transtibial amputation. They found that a 6% increase in volume to participants’ as-prescribed socket could lead to clinically significant changes in quality of fit, comfort, and device satisfaction. Another study conducted by Convery et al.^10^ examined the consistency in cast rectification for transtibial sockets between two experienced prosthetists. In zones of major rectification, such as distal tibia and mid-tibia, it was observed that the mean difference between prosthetists was on average 2 mm with a standard deviation of 1 mm. In our study, the interquartile range among all zones in volume (ranged from 0.6 to 3.9%) and mean depth value (ranged from 0.9 to 2.7 mm) remained relatively small. While it is not a direct comparison, these values were smaller and within a reasonable range compared to previous studies, providing confidence that they could be used in the digital design of transradial sockets.

Furthermore, rectification patterns are often described using angular positions and axial lengths^9–11^. This study introduced a novel 3D unit vector mapping technique to quantitatively depict the average location of each rectification zone within the rectified model. This approach provided a precise description of the location of each rectification zone within 3D space, improving data visualization and comprehension. The vector of each rectification zone exhibited reasonable consistency in all three directions, with the exception of AF and OCF in the proximal–distal direction and OBR in the anterior–posterior direction. However, this issue can be mitigated by integrating existing anatomical landmarks to determine the location of those zones more precisely.

Overall, the findings from this study support the potential for quantitively modelling the transradial socket design process. This opens the door for developing tools for categorizing and predicting socket designs across diverse populations through the application of techniques such as machine learning. A similar approach has been successful for the lower-limb population. Dickinson et al.^7^ previously analyzed and characterized the shape differences among 67 pairs of residual limb and rectified socket models, creating both statistical design models (SDMs) and combined limb shape and design models (SLDMs). These models were able to capture 95% of the population variation in 19 and 4 modes, respectively. This suggests the potential for generalized rectification trends using a computationally efficient method, potentially automating elements for prosthetic design.

This study had several limitations, including a small sample size. Future studies should encompass a larger and more diverse sample population, specifically including participants with acquired amputations and those with long residual limbs. The influences of age, sex and gender on socket design should also be examined with a larger sample size. Further, this study only involved two prosthetists from the same hospital, which increased the likelihood of capturing similar practices, which potentially limits the generalizability of the findings. Involving more prosthetists with varying design approaches in the future would provide a more comprehensive understanding of the rectification process. Nevertheless, this study yielded a more comprehensive, quantitative understanding of the transradial socket-design process, which is a vital initial step towards a more systematic and data-driven approach to socket fabrication. Finally, gaps existing in a lack of quantitative metrics for assessing prosthetic fit and function outcomes. Measures including a Socket Comfort Score exist to rate user-perceived comfort, but not other important aspects of fit (e.g., tissue loading) or function (e.g., suspension)^26^.

## Conclusions

This study is the first of its kind to characterize the design process of the transradial socket by analyzing the shape variation between unrectified and rectified models. By identifying and quantifying rectification features and patterns, this work facilitates the future development of digital socket design using techniques such as those based on machine learning. Similar to research conducted on the lower limb, such models have the potential to categorize and predict socket designs across diverse populations. When combined with prosthetists’ expertise, it can enhance prosthetic design and expedite fabrication, ultimately improving the design and outcomes of transradial prosthetic care.

## Methods

### Data collection

Participants with upper-limb absence at the transradial level were recruited from the client lists of authors E.O. and N.R., who are certified prosthetists with a minimum of 5 years of clinical experience in transradial prosthetic socket design. Inclusion criteria were as follows: (1) presence of transradial limb absence for a minimum of two years and (2) a new prosthetic device that included a socket was required at the time of data collection.

**Figure 6:**
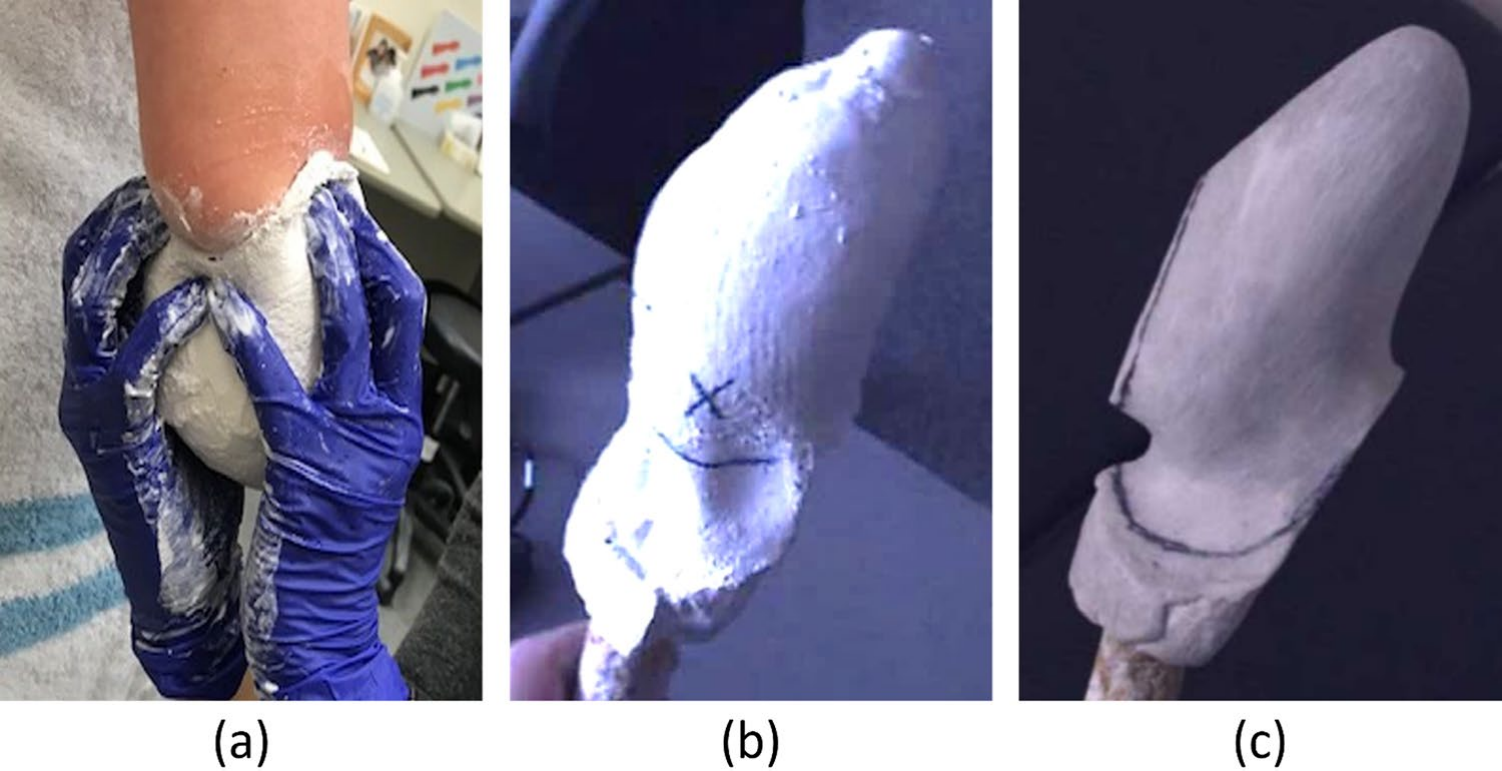
Conventional socket design workflow: (**a**) a negative cast in plaster of Paris bandage of the residual limb; (**b**) unrectified positive plaster model; and (**c**) rectified positive plaster model.

Participants attended a single conventional shape capture session, where casting procedures were carried out by the treating prosthetists, E.O. and N.R. (Fig. 6). A negative cast in regular plaster of Paris bandages of the residual limb was created and subsequently filled with plaster to produce an unrectified positive plaster model for each participant. The unrectified model was then digitally scanned with the Spectra Scanner (Vorum, Vancouver, BC, Canada), a handheld 3D structured light scanner, with a resolution of 0.1 mm. The treating prosthetists manually rectified the model, following the design principles of the three-quarter Northwestern-style transradial socket, to produce a rectified positive plaster model. Same rectification techniques were employed for both the children and adult sockets. The rectified model was also digitized. In addition, upon the completion of the casting process, prosthetists were asked to qualitatively categorize each residual limb on its length, shape, and tissue consistency (Table 4), as this information is important in prosthetic design^7,27^. The study was conducted in accordance with the Declaration of Helsinki and approved by the Research Ethics Board of Holland Bloorview Kids Rehabilitation Hospital (File #19-864; approved on 21 January 2020). Informed consent was obtained from all participants involved in the study. For participants under the age of 18, informed consent was also obtained from their legal guardians.

**Table 4:** Qualitative descriptions of the residual limb.

Factors	Category
Length	Long or short
Shape of residual limb	Conical, round, slender, or with bulbous end
Tissue consistency	Firm, moderate, or soft

### Data processing

**Figure 7:**
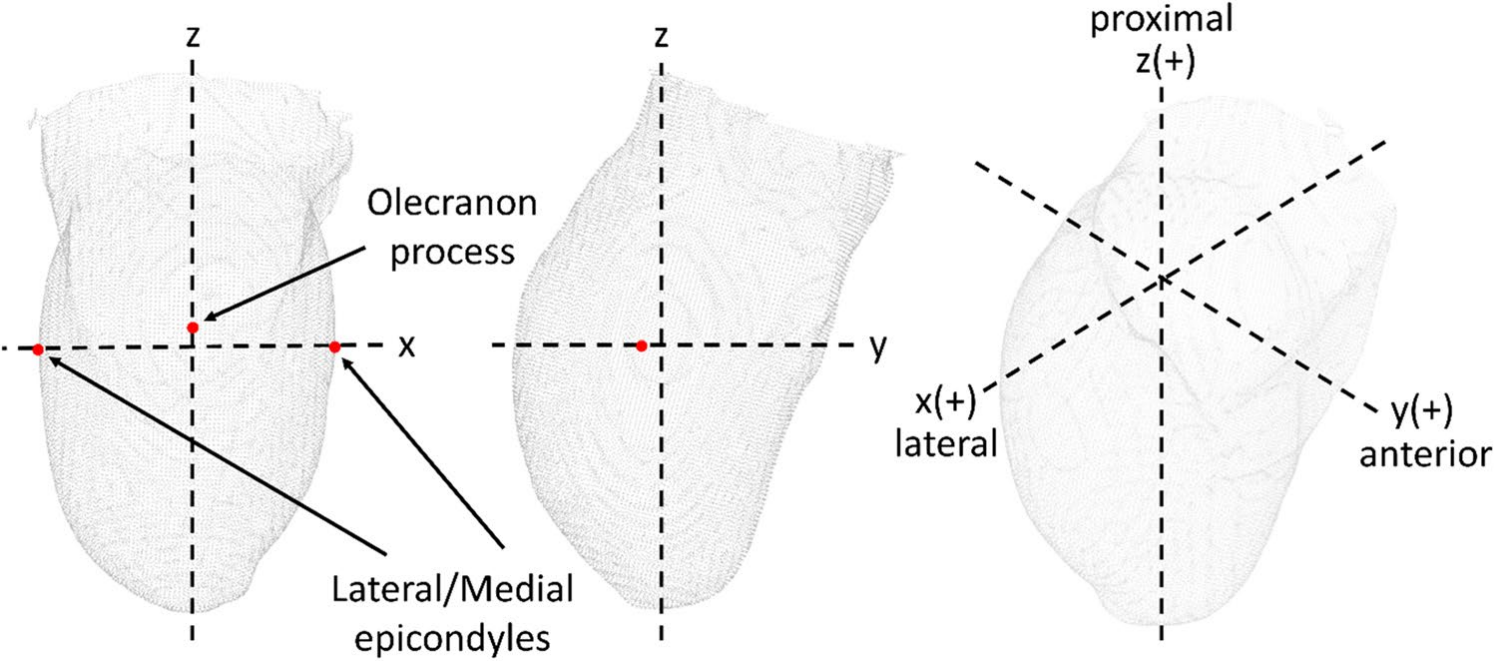
Coordinate convention based on key anatomical landmarks. The x-axis is defined by the lateral and medial epicondyles, the z-axis would pass through the distal end of the model, and the y–z plane is aligned with the olecranon.

Pairs of unrectified and rectified models from each participant were post-processed using the Spectra and Canfit O&P software (Vorum, Vancouver, BC, Canada) and saved as an “STL” file. All right-sided shapes were mirrored. To align and extract the volume of interest of each pair of unrectified and rectified models, a coordinate convention based on key anatomical landmarks was used (Fig. 7). These landmarks include the olecranon process, and lateral and medial epicondyles, as they are known to be invariant (i.e., do not deform during conventional shape capture)^28^. Each pair of models was superimposed, and the iterative closest point algorithm was employed to refine the alignment of these models in CloudCompare 2.11, an open-source 3D point-cloud processing software^29^. Subsequently, two experienced prosthetists (E.O. and N.R.) manually adjusted and verified the alignment^7^.

### Data analysis

To examine the differences between each pair of unrectified and rectified models, global metrics such as the total volume variation and changes in cross-sectional area (CSA) along the length of the model were computed. The volumetric measurement of each model was calculated using Meshmixer 3.5 (Autodesk Inc., San Rafael, CA, USA). In addition, the rectification map and localized rectifications between each pair were also calculated and analyzed^30^.

### Examining the changes in cross-sectional area along the length of models

**Figure 8:**
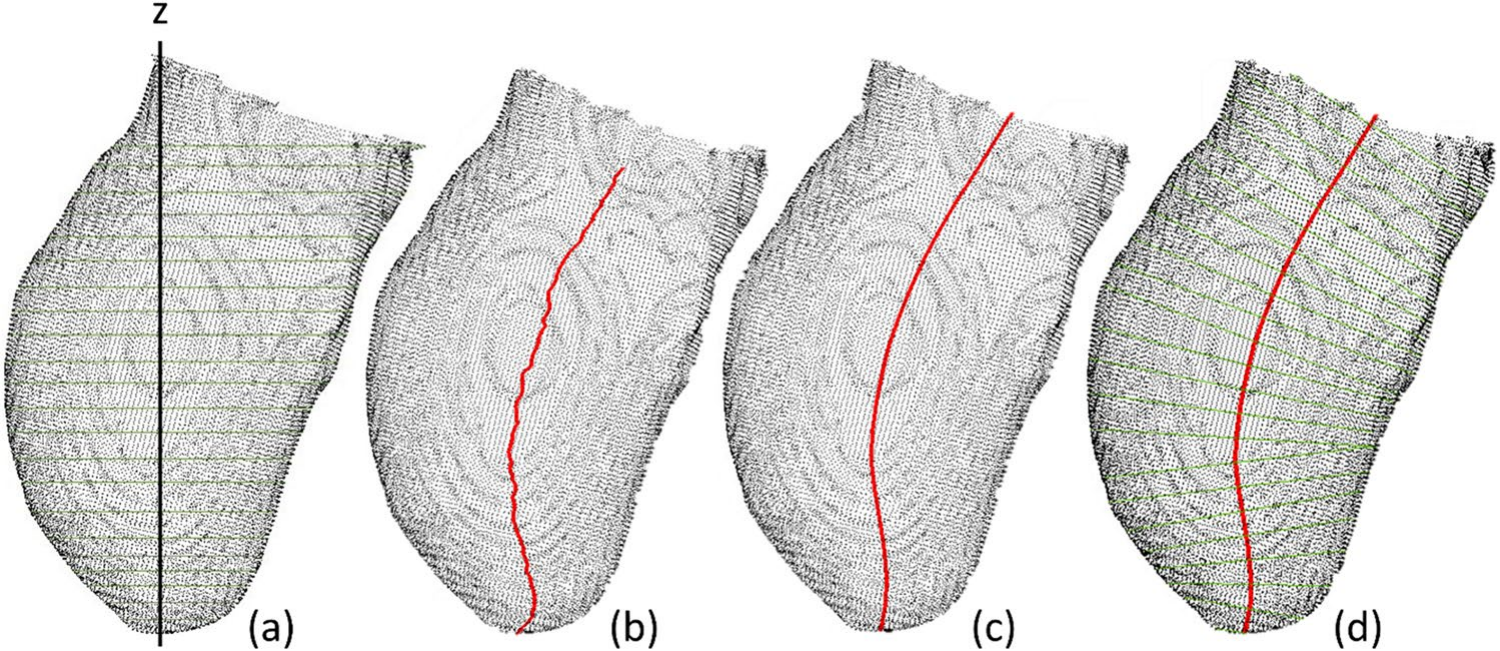
Approximation of the reference axis and slicing: (**a**) slicing in the x–y plane along the z-axis; (**b**) joining the centroids of the aforementioned slices to create a preliminary axis; (**c**) smoothing the axis using a Savitzky–Golay filter; and (**d**) slicing along the reference axis.

Analyzing the changes in CSA along the length of each pair of unrectified and rectified models captures shape rectifications performed by the prosthetist. To do this, a reference axis was established to correlate with the distinct shape and curvature of each residual limb (Fig. 8).

#### Establishing a reference axis

Each unrectified model was first sliced in the x–y plane along the z-axis from the distal end to the proximal cutting plane at intervals of 1% using the AmpScan module developed in Python^31^. The centroid of each slice was then computed and connected longitudinally, resulting in an initial approximation of the reference axis projected onto the sagittal plane^9^. The reference axis was smoothed using a Savitzky–Golay filter within the Scikit-learn module in Python to the mitigate jagged edges^32^. This one-dimensional filter smooths data by applying the least-squares fitting of a polynomial of a specific order to samples in a symmetrical window. A polynomial order of 3 and a window length of 11 were selected based on the preliminary testing and optimization algorithms proposed in previous studies^33,34^.

#### Computing the cross-sectional area for each model

Subsequently, each pair of the unrectified and rectified models along with the corresponding reference axis were imported into CloudCompare 2.11. Both models were then sliced at intervals of 1% along the length of the same reference axis, with each slicing plane perpendicular to the tangent of the reference axis. Finally, the CSA of each slice was computed in Python and the differences between unrectified and rectified models were calculated.

### Identifying and characterizing localized rectification zones

**Figure 9:**
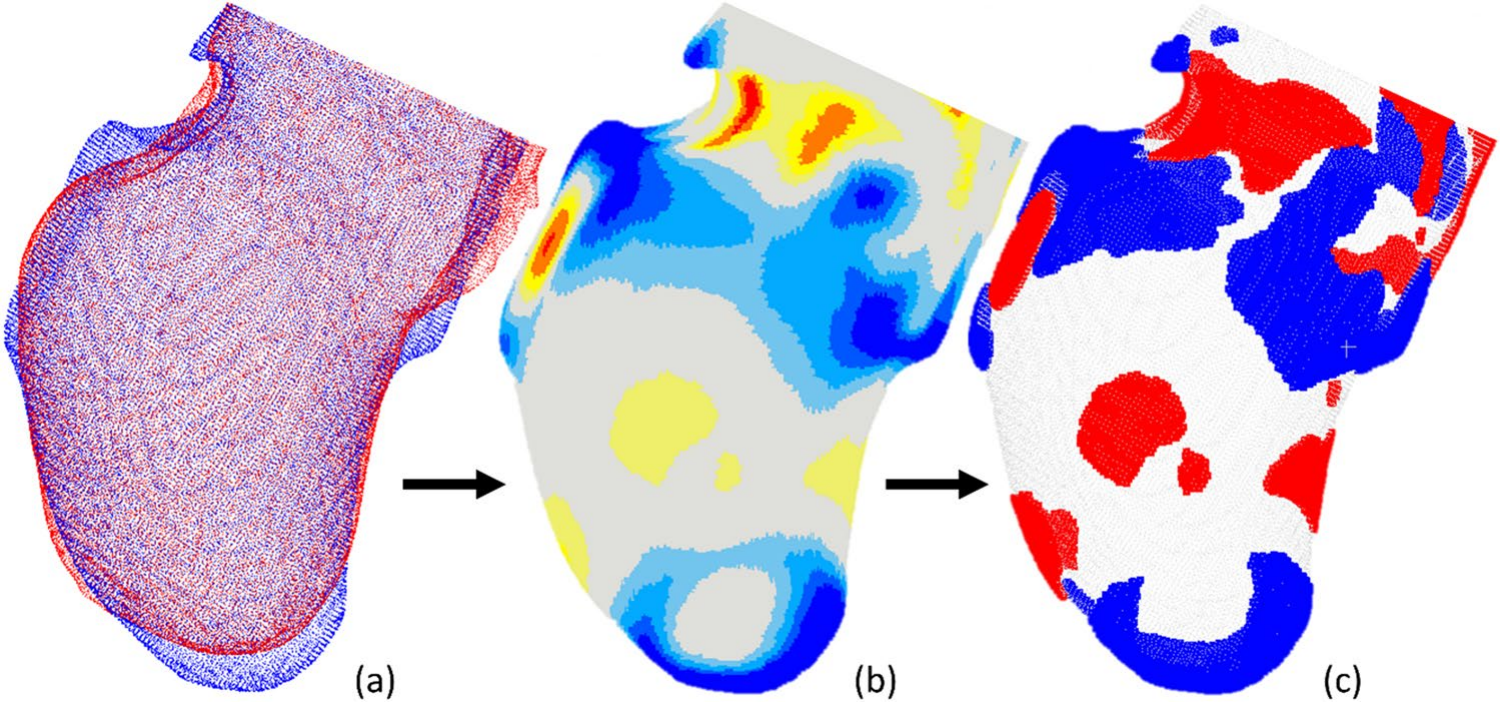
Determining localized rectification zones: (**a**) superposition of the unrectified and rectified cast models; (**b**) generation of a heatmap showing the regions with rectifications; and (**c**) isolation of rectification zones above the threshold (addition and removal of material are denoted in blue and red, respectively).

The characterization of the rectification process was divided into four phases for each pair of the unrectified and rectified models: (1) computing and creating a rectification map; (2) determining threshold values to define localized rectification; (3) quantifying the resulting rectification zones by calculating their surface area, volume, and depth values; and (4) mapping the location of each rectification zone within the residual limb model (Fig. 9).

#### Determining localized rectification zones

A rectification map was used to show the shape differences (degree of rectification) between the 3D unrectified and rectified plaster models^30^. The rectification map was generated by computing the spatial offset between a pair of unrectified and rectified models using CloudCompare 2.11^35^. CloudCompare calculates the distance between corresponding (closest) points between the rectified and unrectified models. Positive depth values denote the addition of plaster while negative depth values indicate the removal of plaster^11^. To identify significant localized rectifications for each rectified/unrectified model pair, a threshold value was set to discern notable surface deviations. To establish these thresholds, the depth values were segregated into two groups—positive and negative—and the mean for each group was then calculated. These mean values served as the thresholds for their respective group. That is, only the points on the surface of the rectified model with a depth value exceeding the mean thresholds were retained. This yielded multiple clusters of points on each model pair. By comparing all model pairs, common clustered groups were identified. The rectification zones were defined using a two-step process: the clustered groups were first examined and correlated with the rectifications of interest listed in relevant literature^17,18^. Subsequently, two experienced prosthetists (E.O. and N.R.) were individually interviewed to verify the identified rectifications of interest on each pair of models^7^.

#### Calculating the surface area, volume, and surface deviations

The rectification zones were characterized by their depth value, surface area, and volume. Since the rectification zones were initially represented as point clouds, a conversion into meshes was necessary prior to the calculation of their surface area and volume. 2D Delaunay triangulation, a widely utilized technique in mesh generation, was employed^36^. The computation of the surface area was then performed using the “shell.area” function within the PyVista module in Python^37^. Subsequently, the surface area was divided by the number of points and then multiplied by the sum of respective depth values within the zone to yield the volume.

#### Mapping the locations of rectification zones

**Figure 10:**
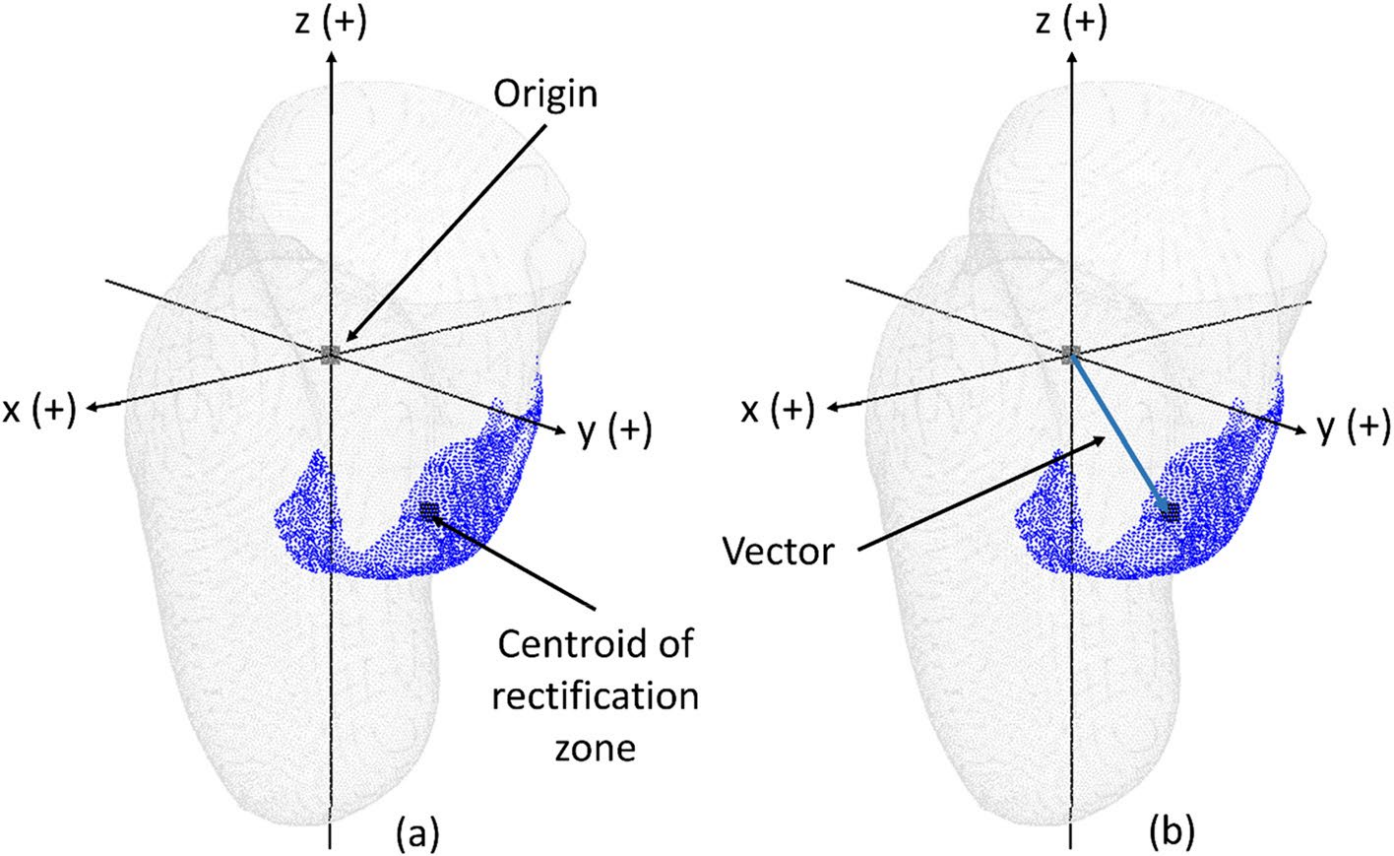
Mapping the locations of rectification zones: (**a**) calculating the centroid of each rectification zone; (**b**) determining the vectors extending from the origin of the model to the centroid of each rectification zone.

To quantitatively map the locations of rectification zones within the rectified model, the centroid of each rectification zone was initially calculated. Subsequently, 3D vectors extending from the origin of the established coordinate system to the centroids of each rectification zone were determined (Fig. 10). Each vector was then converted into a unit vector.

## Data Availability

The data that support the findings of this study are available from the corresponding author upon reasonable request.
